# The Beneficial Effects of Conventional Visual Cues Are Retained When Augmented Reality Glasses Are Worn

**DOI:** 10.1155/2020/4104712

**Published:** 2020-04-07

**Authors:** Sabine Janssen, Jaap de Ruyter van Steveninck, Hizirwan S. Salim, Bastiaan R. Bloem, Tjitske Heida, Richard J. A. van Wezel

**Affiliations:** ^1^Biomedical Signals and Systems Group, MIRA Institute for Biomedical Technology and Technical Medicine, University of Twente, Enschede, Netherlands; ^2^Radboud University Medical Centre, Donders Institute for Brain, Cognition and Behaviour, Department of Neurology, Centre of Expertise for Parkinson & Movement Disorders, Nijmegen, Netherlands; ^3^Department of Biophysics, Donders Institute for Brain, Cognition and Behaviour, Radboud University, Nijmegen, Netherlands

## Abstract

Wearing smart glasses may be distracting and thus annihilate the beneficial effects of cues on freezing of gait in Parkinson's disease. Furthermore, augmented reality cues might be effective in reducing FOG specifically in cueing-responsive patients. We present a single-patient study in which a patient with Parkinson's disease traversed a doorway under different cueing conditions. Wearing augmented reality (AR) glasses did not deteriorate FOG nor affect the beneficial effects of cues. The AR visual cues did not improve FOG. This single-patient study implies that the current design of AR glasses does not stand in the way of the development of augmented reality visual cues. However, the effectivity of augmented reality visual cues remains to be proven.

## 1. Introduction

External cues, such as transverse bars on the floor or the beat of a metronome, can alleviate freezing of gait (FOG) in people with Parkinson's disease (PD) [1–3]. We previously investigated the effects of augmented reality (AR) visual cues on FOG and hypokinetic gait in people with PD but found no improvements [4, 5]. Surprisingly, conventional cues, i.e., real transverse bars on the floor and the beat of a metronome, neither afforded beneficial effects while people wore the AR glasses [4, 5]. One possible explanation is that the rather bulky AR glasses (that were worn throughout the experiment) in effect caused a “dual task” effect and hence inadvertently diverted the participants' attention away from the walking task [4]. Dual tasks are known to deteriorate FOG in PD patients [6–10]. If wearing AR glasses indeed deteriorates FOG, this might annihilate the beneficial effects of both AR and conventional cues. An alternative explanation could be that the participants in these studies were not responsive to cues in general, as this was not a selection criterion.

We hypothesized that wearing AR glasses could have two negative effects: (1) eliciting or worsening FOG; and (2) annihilating the effects of conventional cues. Additionally, we hypothesized that AR cues could alleviate FOG in a cueing-responsive patient.

## 2. Materials and Methods

To test our hypotheses, we tested one person with PD with an established clear response to conventional cues under different cueing conditions. This person was examined with and without wearing AR glasses. The patient was a 63-year-old man who was diagnosed with PD 17 years earlier and who had experienced FOG for 16 years. He had no cognitive impairments and no relevant comorbidities. His FOG partially improved with dopaminergic medication (levodopa equivalent daily dose 2130 mg) but remained bothersome nevertheless. He reported no beneficial or negative effects of bilateral deep brain stimulation of the subthalamic nucleus on his FOG. He successfully used a wide variety of cueing strategies, such as auditory and haptic metronomes, bars and lines on the floor, stepping over a broom, and kicking against a small box on the floor.

The experiment was conducted in a medication OFF state, >12 hours after the last intake of dopaminergic medication. The deep brain stimulator was left switched on. The patient traversed a doorway four times under each of the following conditions: (1) no AR glasses worn, no cues applied (“Control”); (2). The AR glasses worn but switched off (“SG OFF”); (3) AR three-dimensional bars displayed through the AR glasses (“SG AR”); (4) real transverse bars on the floor, no AR glasses worn (“Real bars”); (5) real transverse bars on the floor while wearing AR glasses switched off (“SG real bars”); (6) stepping over a hand-held broom, no AR glasses worn (“Broom”); and (7) kicking against a small box on the floor, no AR glasses worn (“Box”). The AR glasses used were the Microsoft HoloLens (2017, developer version, Microsoft). FOG was annotated from video recordings by two independent experienced raters. The percent time of the trial spent on freezing was compared amongst the different cueing conditions with paired *t*-tests.

## 3. Results and Discussion

**Figure 1:**
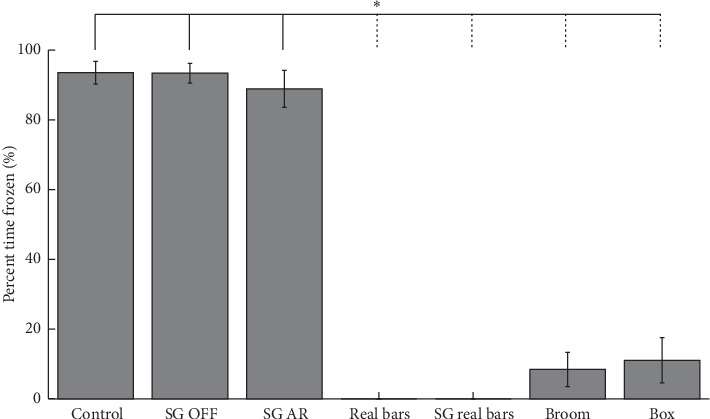
Percent time frozen under different cueing conditions. Bar plot with error bars representing the standard error of the mean. Each of the three conditions on the left (solid vertical bar) shows a statistically significant difference from each of the four conditions on the right (dashed vertical bar) (*p* < 0.05). “Control” = no cues, no AR glasses; “SG OFF” = AR glasses worn and switched off; “SG AR” = AR bars displayed through AR glasses;“Real bars” = real transverse bars on the floor, no AR glasses worn; “SG real bars” = real transverse bars on the floor, AR glasses worn but switched off; “Broom” = stepping over a hand-held broom, no AR glasses worn; and “Box” = kicking against a small box on the floor, no AR glasses worn.

The real bars, broom, and box caused a remarkable and significant improvement in percent time frozen compared to the control condition, confirming that this person was particularly responsive to visual cues (Table 1, Figure 1 and supplementary video 1). Wearing AR glasses did not increase the percent time frozen compared to the control condition, although the percent time frozen was already high (94%) in the control condition. The positive effect of real bars on the percent time frozen was not negatively affected by wearing the AR glasses. The AR visual cues did not improve nor deteriorate FOG (Table 1, Figure 1, and supplementary video 1). This might have been due to the limited field of view of the AR glasses, disrupting the perception of being able to step over the AR bars. Alternatively, the patient's awareness that the AR cues were “not real” might have affected the cues' potency.

## 4. Conclusion

From this single-patient study, we conclude that wearing AR glasses does not worsen FOG nor affect the beneficial effects of conventional cues. This implicates that the current design of AR glasses does not stand in the way of the development of AR visual cues. However, we found no evidence that AR visual cues could improve FOG even in a cueing-responsive patient. Whether these conclusions can be extended to other patients requires further study in a larger cohort. We recommend future studies involving cueing through AR glasses to include control conditions without AR glasses.

## Figures and Tables

**Table 1:** Mean (standard deviation) of duration of trials and FOG episodes and percent time frozen.

	Duration of trials (sec)	Duration of FOG (sec)	Percent time frozen (%)
Control	153,5 (103,4)	146,7 (102,8)	93,5 (5,7)
SG OFF	152,8 (59,5)	144,8 (62,1)	93,3 (5,7)
SG AR	122,2 (56,7)	112,1 (58,6)	88,8 (10,7)
Real bars	4,1 (0,8)	0,0 (0)	0,0 (0)
SG real bars	4,1 (0,5)	0,0 (0)	0,0 (0)
Broom	9,0 (1,6)	0,9 (1,0)	8,4 (9,9)
Box	19,5 (4,7)	2,5 (2,8)	11,1 (11,2)

## Data Availability

All relevant data are available through the supplementary materials.

## Supplementary Materials

Supplementary Materials Legend supplementary video 1. First-person view of the augmented reality three-dimensional transverse bars displayed through the augmented reality glasses. The wearer first moves the head up, looking ahead of the walking track (00 : 00–00 : 03 sec), and then back to just ahead of the feet (00 : 03–00 : 07 sec). Next, the wearer walks forward along the walking track, thereby crossing the augmented reality bars (00 : 07–00 : 19 sec), performs a half turn, and walks back across the same path (00 : 23–00 : 30 sec). Of note: the floor in this demo is different from the floor in the patient measurement. In the patient measurement, there was a carpet with irregular small dots in the room, and a plain grey linoleum floor in the hallway. Legend supplementary video 2. Videos of trials in the experiment under the various conditions: ‘Control' (no cues, no AR glasses) at 0 : 00–00 : 53 sec. ‘SG OFF' (AR glasses worn and switched off) at 00 : 54–02 : 20 sec. ‘SG AR' (AR bars displayed through AR glasses) at 02 : 21–05 : 36 sec. ‘Real bars' (real transverse bars on the floor. no AR glasses worn) at 05 : 36–05 : 42 sec. ‘SG real bars' (real transverse bars on the floor, AR glasses worn but switched off) at 05 : 42–05 : 48 sec. ‘Broom' (stepping over a hand-held broom, no AR glasses worn) at 05 : 49–05 : 55 sec. ‘Box' (kicking against a small box on the floor, no AR glasses worn) at 05 : 56–06 : 14 sec.
